# Emotion Recognition for Human-Robot Interaction: Recent Advances and Future Perspectives

**DOI:** 10.3389/frobt.2020.532279

**Published:** 2020-12-21

**Authors:** Matteo Spezialetti, Giuseppe Placidi, Silvia Rossi

**Affiliations:** ^1^PRISCA (Intelligent Robotics and Advanced Cognitive System Projects) Laboratory, Department of Electrical Engineering and Information Technology (DIETI), University of Naples Federico II, Naples, Italy; ^2^Department of Information Engineering, Computer Science and Mathematics, University of L'Aquila, L'Aquila, Italy; ^3^A^2^VI (Acquisition, Analysis, Visualization & Imaging Laboratory) Laboratory, Department of Life, Health and Environmental Sciences (MESVA), University of L'Aquila, L'Aquila, Italy

**Keywords:** emotion recognition (ER), human-robot interaction, affective computing, machine learning, multimodal data

## Abstract

A fascinating challenge in the field of human–robot interaction is the possibility to endow robots with emotional intelligence in order to make the interaction more intuitive, genuine, and natural. To achieve this, a critical point is the capability of the robot to infer and interpret human emotions. Emotion recognition has been widely explored in the broader fields of human–machine interaction and affective computing. Here, we report recent advances in emotion recognition, with particular regard to the human–robot interaction context. Our aim is to review the state of the art of currently adopted emotional models, interaction modalities, and classification strategies and offer our point of view on future developments and critical issues. We focus on facial expressions, body poses and kinematics, voice, brain activity, and peripheral physiological responses, also providing a list of available datasets containing data from these modalities.

## 1. Introduction

Emotions are fundamental aspects of the human being and affect decisions and actions. They play an important role in communication, and emotional intelligence, i.e., the ability to understand, use, and manage emotions (Salovey and Mayer, 1990), is crucial for successful interactions. Affective computing aims to endow machines with emotional intelligence (Picard, 1999) for improving natural human-machine interaction (HMI). In the context of human-robot interaction (HRI), it is hoped that robots can be endowed with human-like capabilities of observation, interpretation, and emotion expression. Emotions have been considered from three main points of view as follows:

- **Formalization of the robots own emotional state:** the inclusion of emotional traits into agents and robots can improve their effectiveness and adaptiveness and enhance their believability (Hudlicka, 2011). Therefore, the design of robots in the last years has focused on modeling emotions by defining neurocomputational models, formalizing them in existing cognitive architectures, adapting known cognitive models, or defining specialized affective architectures (Cañamero, 2005, 2019; Krasne et al., 2011; Navarro-Guerrero et al., 2012; Reisenzein et al., 2013; Tanevska et al., 2017; Sánchez-López and Cerezo, 2019);
- **Emotional expression of robots:** in complex interaction scenarios, such as assistive, educational, and social robotics (Fong et al., 2003; Rossi et al., 2020), the ability of robots to exhibit recognizable emotional expressions strongly impacts the resulting social interaction (Mavridis, 2015). Several studies focused on exploring which modalities (e.g., face expression, body posture, movement, voice) can convey emotional information from robots to humans and how people perceive and recognize emotional states (Tsiourti et al., 2017; Marmpena et al., 2018; Rossi and Ruocco, 2019);
- **Ability of robots to infer the human emotional state:** robots able to infer and interpret human emotions would be more effective in interacting with people. Recent works aim to design algorithms for classifying emotional states from different input modalities, such as facial expression, body language, voice, and physiological signals (McColl et al., 2016; Cavallo et al., 2018).

In the following, we focus on the third aspect, reporting recent advances in emotion recognition (ER), in particular in the HRI context. ER is a challenging task, in particular when performed in actual HRI, where the scenario could highly differ from the controlled environment in which most of recognition experiments are usually performed. Moreover, the presence itself of the robot represents a bias, since the robot presence, embodiment, and behavior could affect empathy (Kwak et al., 2013), elicit emotions (Guo et al., 2019; Saunderson and Nejat, 2019; Shao et al., 2020), and impact experience (Cameron et al., 2015). For these reasons, we limit our study to articles that perform ER in actual HRI with physical robots. Our aim is to summarize the state of the art and existing resources for the design of emotion-aware robots, discuss the characteristics that are desirable in HRI, and offer a perspective about future developments.

Literature search has been carried out by querying Google Scholar^1^, Scopus^2^, and WebOfScience^3^ databases with basic keywords from HRI and ER domains, and limiting the search to the last years (≥2015). Submitted queries were as follows:

- **Google Scholar:** *allintitle: “human–robot interaction”*| “*human robot interaction”* |*hri emotion*|*affective*, resulting in 80 documents.
- **Scopus:** TITLE ((“human–robot interaction” OR “human robot interaction” OR hri) AND (emotion* OR affective)) OR KEY ((“human–robot interaction” OR “human robot interaction” OR hri) AND (emotion* OR affective)), resulting in 629 documents.
- **WebOfScience:** TI=((“human robot interaction” OR “human–robot interaction” or hri) AND (emotion* or affective)) or AK=((“human robot interaction” or “human–robot interaction” OR hri) AND (emotion* or affective)), resulting in 201 documents.

By using a simple script based on the edit distance between articles titles, we looked for repeated items between search engines results. Note that 425 papers were only on Scopus, 22 on Google Scholar, 11 on WebOfScience, and 38 documents were returned by all the search engines. Between the remaining articles, 150 were both on Scopus and WebOfScience, 16 on Scopus and Google Scholar, and 1 on WebOfScience and Google Scholar. We merged the results in a single list of 664 items. Since we used loose selection queries, resulting articles were highly heterogeneous and most of them were out of the scope of our review. Therefore, we subsequently selected published articles that addressed ER in HRI, reporting significant results with respect to the recent literature, and that *(a)* performed emotion recognition in an actual HRI (i.e., where at least a physical robot and a subject were included in the testing phase), reporting results; *(b)* were focused on modalities that could be acquired, during HRI, by using both robot's embedded sensors or external devices: facial expression, body pose and kinematics, voice, brain activity, and peripheral physiological responses; and *(c)* relied on either discrete or dimensional models of emotions (see section 2.1). This phase allowed to select 14 articles. During the process, however, we also looked at the references of the selected paper in order to find other works that fit our inclusion criteria. In this way, 3 articles were added to this review. Finally, we organized the resulting articles by considering modalities and emotional models.

## 2. State of the Art

### 2.1. Emotional Models

A key concept in ER is the model used to represent emotions, since it affects the formalization of the problem and the definition and separation of classes. Several models have been proposed for describing emotions, as reported in Table 1. The main distinction is between categorical models, in which emotions consist of discrete entities associated with labels, and dimensional models, in which emotions are defined by continuous values of their describing features, usually represented on axes.

**Table 1:** Emotional models.

**Model**	**Type**	**Description**
Ekman (Ekman, 1992)	Categorical	Six basic emotions (anger, disgust, fear, happiness, sadness, and surprise) characterized by distinctive universal signals, and physiology
Tomkins (Tomkins, 2008)	Categorical	Seven affects organized in low/high intensity couples (interest-excitement, enjoyment-joy, surprise-startle,distress-anguish, anger-rage, fear-terror, shame-humiliation), plus disgust, and *dismell*
Valence/Arousal (VA) (Russell, 1980)	Dimensional	Emotions represented over a two-dimensional circular space: axes describe the valence and arousal
Pleasure/Arousal/Dominance (PAD) (Mehrabian, 1996)	Dimensional	Emotions described with three dimensions:pleasure, arousal, and dominance
3D hypercube (Trnka et al., 2016)	Dimensional	Emotions described with three dimensions: valence, intensity, controllability, and utility
Plutchik wheel of emotions (Plutchik and Kellerman, 2013)	Hybrid	Eight primary emotions differentiated by levels of intensity

*For each entry the type of model and a brief description are reported*.

It is hard to say which model is better for representing emotions, the debate is still open and it is strictly related to the nature of emotions, with a lack of consensus (Lench et al., 2011; Lindquist et al., 2013). Some scholars claim that emotions are discrete natural kinds associated with categorically distinct patterns of activation at the level of autonomic nervous system (Kragel and LaBar, 2016; Saarimäki et al., 2018). Others point out intra-emotion differences and the overlap of different emotions with respect to observed behavior end autonomous activity (Siegel et al., 2018). Since the purpose of this work is not to support one or the other thesis, we step back from this discussion and focuses on the usability of the models. From the point of view of the recognition process, the possibility to identify distinct emotions is, ideally, the simplest method. Unfortunately, as the number of emotions considered increases, it becomes more difficult to distinguish between classes. On the other hand, despite the usefulness of obtaining information about features of emotions (e.g., valence and arousal), a small number of dimensions could lead to an over-simplification and to an “overlap” of different emotions that share similar values of features (Liberati et al., 2015). For this reason, the choice of informative and possibly uncorrelated dimensions is critical (Trnka et al., 2016). Datasets are often annotated by using both categorical and dimensional models, but it could be observed that those employing discrete models do not ever use the same labels, that is annotated emotions differ both in number and names. Conversely, several dimensional annotated datasets share a common valence-arousal (VA) (Russell, 1980) representation, which allows to compare and merge data from different datasets, in the worst case by ignoring additional axes. When annotating a dataset, a good practice is to provide at least the VA labels.

Table A1 in Supplementary Material reports a non-comprehensive list of datasets that can be used to train and test ER approaches.

### 2.2. Facial Expressions

A natural way to observe emotions is the analysis of facial expressions (Ko, 2018). Conventional facial emotion recognition (FER) systems aim to detect the face region in images and to compute geometric and appearance features, which are used to train machine learning (ML) algorithms (Kumar and Gupta, 2015). Geometric features are obtained by identifying facial landmarks and by computing their reciprocal positions and action units (AUs) (Ghimire and Lee, 2013; Suk and Prabhakaran, 2014; Álvarez et al., 2018), while appearance-based features are based on texture information (Turan and Lam, 2018).

In recent years, deep learning (DL) approaches have emerged. DL aims to develop *end-to-end* systems to reduce the dependency from hand-crafted features, pre-processing, and feature extraction techniques (Ghayoumi, 2017). Notably, convolutional neural networks (CNNs) have been proven to be particularly efficient in this task (Mollahosseini et al., 2017; Zhang, 2017; Refat and Azlan, 2019).

When dealing with video-clips, also the temporal components of data can be exploited. In traditional FER, this is usually accomplished by including in the features vector information about landmarks displacement between frames (Ghimire and Lee, 2013). In DL approaches, temporal information is handled by means of specific architectures and layers, such as recurrent neural network (RNN) and long-short term memory (LSTM) (Ebrahimi Kahou et al., 2015).

In the context of HRI, FER has been performed through conventional and DL approaches.

#### 2.2.1. Discrete Models

In Faria et al. (2017), an emotion classification approach, based on simple geometrical features, was proposed. Given the position of 68 facial landmarks, their Euclidean distances and the angles of 91 triangles formed by landmarks were considered. A probabilistic ensemble of classifiers, namely dynamic Bayesian mixture model (DBMM), was employed using linear regression (LR), support vector machine (SVM), and an random forest (RF), combined in a probabilistic weighting strategy. The proposed approach was tested both on Karolinska Directed Emotional Faces (KDEF) (Lundqvist et al., 1998) and during HRI for recognizing 7 discrete emotions. In particular, for HRI test, the humanoid robot NAO was programmed either to react to the recognized emotion. The overall accuracy on the KDEF dataset was 85%, while in actual HRI it was 80.6%. It is to note that the test performed on KDEF was limited to images of frontal or ±45° orientation of the faces.

In Chen et al. (2017), the authors proposed a method for real-time dynamic emotion recognition according to facial expression and emotional intention understanding. In particular, emotion recognition was performed by using a dynamic model of AUs (Candide3-based dynamic feature point matching) and an algorithm implementing fuzzy rules for each of the 7 basic emotions considered. Experiments were conducted on 30 volunteers experiencing the scenario of drinking at bar. One of the 2 employed mobile robot was used for emotion recognition, achieving 80.48% of accuracy.

Candide3-based features were also adopted in Chen et al. (2019). Here, the authors proposed an adaptive feature selection strategy based on the plus-L minus-R selection (LRS) algorithm in order to reduce the model dimensionality. Classification was performed with a set of k-nearest neighbors (kNN) classifiers, integrated into AdaBoost with direct optimization framework. The proposed approach was tested both on the JAFFE dataset (Lyons et al., 1998) and on data acquired by a mobile robot, equipped with a Kinect. In the latter experiment, the proposed method achieved an average accuracy of 81.42% in the classification of 7 discrete emotions.

In Liu et al. (2019), FER was performed by combining local binary pattern (LBP) and 2D Gabor wavelet transform for feature extraction and by training an extreme learning machine (ELM) for the classification of basic emotion. Experiments were conducted both on public datasets [JAFFE (Lyons et al., 1998), CK+ (Lucey et al., 2010)], and during actual HRI as part of a multimodal system setup (Liu et al., 2016). In the latter case, the method was able to recognize between 7 emotions with an overall accuracy of 81.9%.

Histogram of oriented gradients (HOG) and LBP were used as features descriptors in Reyes et al. (2020), where a SVM was trained to classify 7 discrete emotions. The system was initially fed with images from extended Cohn-Kanade (CK+) dataset, but was fine-tuned, by adding batches of different sizes of local images (i.e., facial images of participants acquired during the test). Classification of data acquired during the interaction with the robot NAO achieved 87.7% of accuracy.

Reported results suggest that FER systems designed for HRI can be developed by using several features and decision strategies. However, differences between HMI and HRI arise. In HMI scenarios, FER is in general easier: the position of the face with respect to the camera is more constrained, the user is close to the camera and the environment conditions do not change abruptly. Due to these differences, it is preferable to train FER system on data *in-the-wild* or during real interaction with robots. Moreover, it could be useful to endow robots with the capability of recognizing emotions not just from facial information, but also from contextual and environmental data (Lee et al., 2019). Future developments of FER will probably depend also on emerging technologies: in the last decades, there was a rapid development of relatively cheap depth cameras (RGB-D sensors), and thermal cameras. The work by Corneanu et al. (2016) offers a comprehensive taxonomy of FER approaches based on RGB, 3D, and thermal sensors. For example, 3D information can improve face detection, landmarks localization, and AUs computation (Mao et al., 2015; Szwoch and Pieniażek, 2015; Zhang et al., 2015; Patil and Bailke, 2016).

### 2.3. Thermal Facial Images

Changes in the affective state produce the redistribution of the blood in the vessels, due to vasodilatation/vasoconstriction and emotional sweating phenomena. Infra-red thermal cameras can detect these changes, since they cause variations in skin temperature (Ioannou et al., 2014). Therefore, thermal images could be used to perform ER (Liu and Wang, 2011; Wang et al., 2014). Usually, this is done by considering temperature variations of specific regions of interest (ROIs), e.g., tip of the nose, forehead, orbicularis oculi, and cheeks.

#### 2.3.1. Discrete Models

In Boccanfuso et al. (2016), 10 subjects played a trivia game with a MyKeepon robot behaving to induce happiness and anger and watched emotional video clips selected to elicit the same emotions. RGB and thermal facial images were acquired, together with galvanic skin response (GSR) signal. In particular, the thermal trends of 5 ROIs were analyzed by combining principal component analysis (PCA) and logistic regression, achieving a prediction success of 100%.

In Goulart et al. (2019b), a system for the classification of 5 emotions during the interaction between children and a robot was proposed. A mobile robot (N-MARIA) was equipped RGB and thermal camera that were used to locate and acquire thermal information of 11 ROIs, respectively. Statistical features were computed for each ROI and multiple combinations of feature reduction techniques and classification algorithms were tested over a database of 28 developing children (Goulart et al., 2019a). A PCA+linear discriminant analysis (LDA) system, trained and tested on the database (accuracy 85%), was used to infer the emotional responses of children during the interaction with N-MARIA, with results consistent with self reported emotions.

Performing ER from thermal images in actual HRI is still a challenge due to the constraints (other than those that affect simple FER) that are not adaptable to all real-life scenarios, first of all the necessity to maintain a stable environmental temperature. However, recent results show that thermal images have the potential to facilitate HRI (Filippini et al., 2020).

### 2.4. Body Pose and Kinematics

As facial expressions, body posture, movements, and gestures are natural and intuitive ways to infer the affective state of a person. Emotional body gesture recognition (EBGR) has been widely explored (Noroozi et al., 2018). In order to take advantage of information conveyed by static or dynamic cues, an EBGR system has to model the body position from input signals, usually RGB data, depth maps, or their combination. The first step of the canonical recognition pipeline is the detection of human bodies: literature offers several approaches for addressing the problem (Ioffe and Forsyth, 2001; Viola and Jones, 2001; Viola et al., 2005; Wang and Lien, 2007; Nguyen et al., 2016). Then, the pose of the body has to be estimated, by fitting an a priori defined model, typically a skeleton, over the body region. This task could be performed either by solving an inverse kinematic problem (Barron and Kakadiaris, 2000) or by using DL, if a large amount of skeletonized data are available (Toshev and Szegedy, 2014). Features could include absolute or reciprocal positions and orientations of limbs, as well as movement information such as speed or acceleration (Glowinski et al., 2011; Saha et al., 2014). Classification can be performed either by traditional ML or deep learning (Savva et al., 2012; Saha et al., 2014).

#### 2.4.1. Discrete Models

An interesting approach was proposed in Elfaramawy et al. (2017), where neural network architecture was designed for classifying 6 emotions from body motion patterns. In particular, the classification was performed by grow when required (GWR) networks, self-organizing architectures able to grow nodes whenever the network does not sufficiently match the input. Two GWR network learned samples of pose and motion and, subsequently, a recurrent variant of GWR, namely gamma-GWR, to take in account temporal context. The dataset, that included 19 subjects, was collected by extending a NAO robot with a depth camera (Asus Xtion) and using a second NAO located on the side to allow acquisition from two points of view. Subjects performed body motions related to the emotions, elicited by the description of inspiring scenarios. Pose features (positions of joints) and motion features (the difference in pose features between consecutive frames) were considered. The system achieved an overall accuracy of 88.8%.

#### 2.4.2. Dimensional Models

In Sun et al. (2019), the authors proposed the local joint transformations for describing body poses, and a two-layered LSTM for estimating the emotional intensity of discrete emotions. The authors tested the system over the Emotional Body Motion Database (Volkova et al., 2014) by considering the intensity as the percentage of correctly perceived segments for each scenario. Pearson Correlation Coefficient (PCC) between the ground truth and the estimated intensity was 0.81. In real HRI experiments with a Pepper robot, the system enabled the robot to sense subjects' emotional intensities effectively.

Summarizing these results, we can say that body poses and movements are excellent to convey emotional cues and that EBGR systems can be successfully employed in HRI scenarios. Moreover, the fact that FER and EBGR rely on the same sensors (RGB, depth cameras) would allow to take advantages of both modalities.

### 2.5. Brain Activity

Inferring the emotional state brain activity represents a challenging and fascinating possibility, since having access to the cerebral activity would allow to avoid any filter, voluntary or not, that could interfere with the ER (Kappas, 2010). Several measurement systems could be used for acquiring brain activity. Among them, electroencephalography (EEG) is characterized by high temporal resolution, is portable, easy to use, and not expensive. Moreover, it has proven to be suitable for brain monitoring also in HMI applications (Lahane et al., 2019). Consumer-grade devices, although not accurate enough for neuroscience research and critical control tasks, have been reported to be a feasible choice for applications such as affective computing (Duvinage et al., 2013; Nijboer et al., 2015; Maskeliunas et al., 2016).

ER by EEG has been widely explored in the literature (Alarcao and Fonseca, 2017; Spezialetti et al., 2018). Most commonly used features can be roughly classified by the domain from which they are extracted (time, frequency, time–frequency). Time domain features include statistical values, Hjorth parameters, fractal dimension (FD), and high order crossing (HOC) (Jenke et al., 2014). Frequency analyses in EEG are very common, also because it is known the association between frequency bands of EEG signal and specific mental tasks. The most intuitive and used frequency feature is band power, but other measures, such as high order spectra (HOS) have been also employed (Hosseini et al., 2010). Time–frequency analysis aims to observe the frequency content of the signal, without losing the information about its the temporal evolution. Among others, wavelet transform (WT) has demonstrated to be particularly suited for analyzing non-stationary signals such as EEG (Akin, 2002). Previously listed features are generally computed in a channel wise manner, but also the topography of EEG signals can be taken into account. Since frontal EEG asymmetry has been proved to be involved in emotional activity (Palmiero and Piccardi, 2017), asymmetry indices have been often employed in emotion recognition.

Some interesting works in traditional literature (Ansari-Asl et al., 2007; Petrantonakis and Hadjileontiadis, 2009; Valenzi et al., 2014) were devoted to test which classification approaches, features, and channel configurations are more suited for EEG-based ER. Results from these studies suggest two significant points. First, the emotional state of subjects can be inferred with quite good accuracy by EEG. Second, using a reduced set of channels and commercial-grade devices still allows to preserve an adequate level of accuracy. The latter point is critical, since in most of the HRI scenarios, the EEG equipment should be worn continuously for long periods and while moving. Light, easy-to-mount devices would be preferable to research-grade hardware. Until 2017 (Al-Nafjan et al., 2017), the most adopted classification approach was SVM, often in conjunction with power spectral density (PSD)-based features. However, deep learning approaches are standing out also in this domain, showing the potential to outperform traditional ML techniques (Zheng and Lu, 2015; Li et al., 2016; Wang et al., 2019). However, to the best of our knowledge, few works have addressed EEG ER in real HRI scenarios.

#### 2.5.1. Dimensional Models

In Shao et al. (2019), the authors employed the Softbank Pepper robot as an autonomous exercise facilitator to encourage the user during the physical activity that can autonomously adapt its emotional behaviors on the basis of user affect. In particular, valence detection was performed by analyzing EEG from a commercial-grade device. Selected features were PSD and frontal asymmetry. Among 6 classifiers, a neural network (NN) achieved the highest accuracy over a dataset from 10 subjects obtained by inducing emotions with pictures and videos. When employed in a real HRI scenario, the robot was able to correctly recognize the valence (5 levels) for 14 of the 15 subjects.

In Shao et al. (2020), the authors proposed a novel paradigm for eliciting emotions by directly employing non-verbal communication of the robot (Pepper), in order to train a detection model with data from actual HRI. The elicitation methodology was based both on music and body movements of the robot and aimed to elicit two types of affects: positive valence and high arousal, and negative valence and low arousal. EEG was acquired by a 4-channel headset in order to extract PSD and frontal asymmetry features and feed an NN and an SVM. The affect detection approach was tested on 14 subjects for valence and 12 for arousal, obtaining an overall accuracy of 71.9 and 70.1% (valence), and 70.6 and 69.5% (arousal) using NN and SVM, respectively.

### 2.6. Voice

Everyday experiences tell us that the voice, as well as facial expressions, is an informative channel about our interlocutor emotions. We have a natural ability to infer the emotional state underlying the semantic content of what the speaker is saying. Changes in emotional states correspond to variations of organs' features, such as larynx position and vocal fold tension, thus in variations of the voice (Johnstone, 2001). In HRI, automatic acoustic emotion recognition (AER) has to be performed in order to allow robots to perceive human vocal affect. Usually, AER does not examine speech and words in the semantic sense, instead it analyzes variation with respect to the neutral speak of prosody (e.g., pitch, energy, and formants information), voice quality (e.g., voice level and temporal structures), and spectral (e.g., cepstral-based coefficients) features. Features can be extracted either locally, segmenting the signal in frames, or globally, considering the whole utterance. In traditional ML approaches, feature extraction is followed by classification, performed mostly by hidden Markov model (HMM), Gaussian mixture model (GMM), and SVM (El Ayadi et al., 2011; Gangamohan et al., 2016). Also in the AER field, deep learning approaches have rapidly emerged, providing *end-to-end* mechanisms in contrast with those based on hand-crafted features and demonstrating that they can perform well-compared with traditional techniques (Khalil et al., 2019). Employed models include deep Boltzmann machine (DBM) (Poon-Feng et al., 2014), RNN (Lee and Tashev, 2015), deep belief network (DBN) (Wen et al., 2017), and CNN (Zheng et al., 2015).

#### 2.6.1. Discrete Models

In Chen et al. (2020), two-layer fuzzy multiple random forest (TLFMRF) was proposed for speech emotion recognition. Statistic values of 32 features (16 basic features and their derivative) were extracted from speech samples. Then, clustering by fuzzy C-means (FCM) was adopted to divide the feature data into different subclasses to address differences in identification information such as gender and age. In TLFMRF, a cascade of RF was employed for improving the classification between emotions that are difficult to distinguish. The approach was tested in the classification of six basic emotions from short utterances, spoken by 5 participants in front of a mobile robot. Average accuracy was 80.73%.

### 2.7. Pheripheral Physiological Responses and Multimodal Approaches

Emotions affect body physiology, producing significant modifications to hearth rate, blood volume pressure (BVP), respiration, skin conductivity, and temperature (Kreibig, 2010), which can contribute to predict the emotional state of a person. A large effort has been made for developing datasets and techniques for ER, often by considering multiple signal sources together (Koelstra et al., 2011; Soleymani et al., 2011; Chen et al., 2015; Correa et al., 2018). Beside the accuracy obtained with just peripheral signals, these works show that the fusion of multiple modalities can outperform single modality approaches, therefore they can be useful sources of information for improving multimodal system performance. Moreover, the rapid development and mass production of consumer grade devices, such as smartband and smartwatch (Poongodi et al., 2020), will facilitate the integration of these signals in most of HRI systems. For example, in Lazzeri et al. (2014) a multimodal acquisition platform, including a humanoid robot capable of expressing emotions, was tested in a social robot-based therapy scenario for children with autism. The system included audio and video sources, together with electrocardiogram (ECG), GSR, respiration, and accelerometer, that are integrated in a sensorized t-shirt, but the platform was designed to be flexible and reconfigurable in order to connect with various hardware devices. Another multimodal platform was described in Liu et al. (2016). It was a complex multimodal emotional communication based human–robot interaction (MEC-HRI) system. The whole platform was composed of 3 NAO robots, two mobile robots, a workstation, and several devices such as a Kinect and an EEG headset, and it was designed to allow robots to recognize humans' emotions and respond in accordance to them, basing on facial expression, speech, posture, and EEG. The article does not report numerical results of multimodal classification in tested HRI scenarios, but modules achieved promising results when tested on benchmark datasets (Liu et al., 2018a,b) or in single-modality HRI experiments (Liu et al., 2019).

At present, several studies have employed single peripheral measures for ER in HRI (McColl et al., 2016), but the majority focused on narrow aspects of ER (e.g., level of stress or fatigue), instead of referring to a broader emotional model.

#### 2.7.1. Discrete Models

One of the highest accuracy results, we found in the literature, was Perez-Gaspar et al. (2016). Here, the authors developed a multimodal emotion recognition system that integrated expressions and voice patterns, based on the evolutionary optimization of NN and HMM. Genetic algorithms (GAs) were used to estimate the most suitable structures for ANNs and HMMs for modeling of speech and visual emotional features. Speech and visual information were managed separately by two distinct modules and a decision level fusion was performed by averaging the output probabilities from different modalities of each class. The system was trained on a dataset of Mexican people, containing pictures from 9 subjects and speech samples from 8 subjects. Four basic emotions were considered (anger, sadness, happiness, and neutral). Live tests were performed with 10 unseen subjects that interacted with a graphical interface and 97% of accuracy was reported. Finally, in all HRI experiments (dialogue with a Bioloid robot), the robot speech was consistent with the emotion shown by the users.

In Filntisis et al. (2019), the authors proposed a system that hierarchically fuses body and facial features from images based on the simultaneous use of residual network (ResNet) and a deep neural network (DNN) to analyze face and body data, respectively. The system was not incorporated into a robot, but tested on a database containing images of children interacting with two different robots (Zeno and Furhat) in a game in which they had to express 6 basic emotions. Classification accuracy was 72%.

An interesting approach was proposed in Yu and Tapus (2019) for multimodal emotion recognition from thermal facial images and gait analysis. Here, interactive robot learning (IRL) was proposed to take advantage of human feedback obtained by the robot during HRI. First, two RF models for thermal images and gait were trained separately on a dataset of 15 subjects labeled with 4 emotions. Computed features included PSD of 4 joints angles and angular velocity for gait and mean and variance of 3 ROIs for thermal images. A decision level fusion was performed based on weights computed from the confusion matrices of the two RF classifiers. In the proposed IRL, during the interaction with the robot, if the predicted emotion does not correspond to the human feedback, the gait and the thermal facial features were used to update the emotion recognition models. The online test included emotion elicitation by movie, followed by gait and thermal images acquisition, and involve a Pepper robot. Results showed the IRL can improve the classification accuracy from 65.6 to 78.1%.

#### 2.7.2. Dimensional Models

Barros et al. (2015) presented a neural architecture, named Cross-Channel CNN for multimodal data extraction. Such network is able to extract emotions' features based on face expression and body motion. Among different experiments, the approach was tested on a real HRI scenario. An iCub robot was used to interact with a subject and presented one emotional state (positive, negative, or neutral). The robot recognized the emotional state and gave feedback by changing its mouth and eyebrow LEDs, with an average accuracy of 74%.

In Val-Calvo et al. (2020), an interesting analysis of the possibilities of ER in HRI was performed by using facial images, EEG, GSR, and blood pressure. In a realistic HRI scenario, a Pepper robot dynamically drives subjects' emotional responses by story-telling and multimedia stimuli. Acquired data (from 16 participants) was labeled with the emotional score that each subject self reported using 3 levels of valence and arousal. Classification experiments were conducted, together with a population-based statistical analysis. Facial expression estimation is achieved by a CNN strategy. The model was trained on FER2013 (Goodfellow et al., 2013) database in order to map facial images in 7 emotions, grouped into 3 levels of valence. Three independent classifications were used for estimating valence from EEG and arousal from BVP and GSR. The classification process was carried using a set of 8 standard classifiers and considering statistical features of the signals. Achieved accuracy results obtained on both emotional dimensions were higher than 80% on average.

## 3. Discussion

As it is possible to observe by our brief summary about the state of the art, ER is feasible by collecting different kinds of data. Some modalities have been widely explored, both in a broader HMI context and specifically for HRI (FER, EBGR), others should be deeper investigated because, at present, ER has not been tested enough in HRI applications (EEG) or because existing HRI field tests are focused on narrow aspects of emotions (peripheral responses). In our opinion, all the considered modalities represent promising information sources for future developments: innovative and accessible technologies, such as depth cameras, consumer-grade EEG, and smart devices, together with advances in ML will lead to rapid developments of emotions-aware robots. Nevertheless, emotion recognition is currently still a challenge for robots due to the necessity of reliability of results, to provide a trustworthy interaction, and the time constraints required to account for the recognized emotion into the adaptation of the robot behavior. Moreover, many of the used dataset came from general HMI research and so are not suited for emotion recognition in real settings. There is still the need of dataset from real HRI. Indeed, the visual field of view of the robot may not be aligned to the images stored in the dataset (i.e., form face-to-face interaction), the perceived sound may be affected by noise of the ego-motion of the robot, and robot movements may even occlude its field of view. In this perspective, multimodal systems will have a key role by improving the performances of ER with respect to single-modalities approaches, and ML methods and DL architectures have to be developed to deal with heterogeneous data. Particular attention has to be paid on data used to train and test ER: HRI presents some critical and challenging aspects that could make data collected in controlled environments or from different contexts, unsuitable for real HRI applications. However, recently published datasets have the advantage of containing data collected from a large number of sensors. This is a valuable feature, since it will allow to develop features-level fusion approaches for multimodal ER.

## Author Contributions

SR conceived the study. MS contributed to finding the relevant literature. All authors contributed to manuscript writing, revision, and read and approved the submitted version.

## Conflict of Interest

The authors declare that the research was conducted in the absence of any commercial or financial relationships that could be construed as a potential conflict of interest.

## Footnotes

^1^scholar.google.com

^2^www.scopus.com

^3^apps.webofknowledge.com
