# Supplementary material for: Emotion Recognition for Human-Robot Interaction: Recent Advances and Future Perspectives
Source: Front Robot AI. 2020 Dec 21;7:532279. doi: 10.3389/frobt.2020.532279 (PMC7806093; doi:10.3389/frobt.2020.532279)
Supplement: Supplementary file 1 [file Data_Sheet_1.pdf]

frontiers

Supplementary Material

APPENDIX A - DATASETS FOR EMOTION RECOGNITION

The following table aims to provide a list of available datasets for emotion recognition from different sources. For each entry, we indicate (if possible): available data sources, number of participants, emotional labels, methods for inducing emotions. In order to make the table available and constantly updated, we provide a GitLab repository [1]. Please refer to that repository for downloading the newest version (with source files) and for reporting missing references.

[1] https://gitlab.com/matteo.spezialetti/datasets-for-emotion-recognition

Dataset	Data	Subjects	Labeling (S=self-assessment, E=external annotation)	Emotion Induction	Notes
KDEF (Lundqvist et al., 1998)	Facial images	70	Discrete (E): 7 labels	Poses	-
JAFFE (Lyons et al., 1998)	Frontal images	10	Discrete (E): 6 labels	Poses	-
Database of German Emotional Speech (Burkhardt et al., 2005)	Speech	10	Discrete (E): 7 labels; Dimensional (E): Valence, Arousal, Dominance	Simulated emotions	-
BU-3DFE (Yin et al., 2006)	3D shape models; 2D face textures	100	Discrete (S): 6 labels (4 intensities) + neutral	Poses	-
Bosphorus (Savran et al., 2008)	3D shape models; 2D face textures	105	Discrete (S): 9 labels	Poses	AUs annotations
IEMOCAP (Busso et al., 2008)	Motion Capture; Speech;	10	Discrete (E): 9 labels; Dimensional (E): Valence, Arousal, Dominance	Performing in emotional scenarios	-
CINEMO (Rollet et al., 2009)	Speech;	50	Discrete (E): 16 labels + 6 macro classes; Dimensional (E): Valence, Activation, Control Suddenness Intensity Naturalness	Dubbing movies	-
JEMO (Brendel et al., 2010)	Speech;	39	Discrete (E): 4 labels	Emotions detection game	-
NIMITEK (Gnjatovic and Rosner, 2010)	Videos; Speech; Desktop;	10	Discrete (E): 8 labels	Performing tasks on PC	German language
NVIE (Wang et al., 2010)	Videos; Thermal maps	215	Dimensional (S and E): Valence, Arousal Discrete (S and E): 6 labels	Emotional videos; Poses	-
Inter-ACT (Castellano et al., 2010)	Short videos (6s): face (frontal, lateral), full body; contextual game data	8 children	Dimensional (E): Valence, Interest	-	HRI with iCat
Extended Cohn-Kanade (CK+) (Lucey et al., 2010)	Frame sequences (9-60 frames): face (frontal, lateral)	210	Discrete (S): 6 labels	-	FACS annotations (?)

DEAP<br>(Koelstra et al., 2011)	EEG;<br>GSR;<br>Respiration;<br>Skin temperature;<br>ECG;<br>Blood volume;<br>EMG;<br>Frontal videos	32	Dimensional (S): Valence,<br>Arousal,<br>Dominance,<br>Liking	Music videoclips	-
MAHNOB HCI<br>(Soleymani et al., 2011)	EEG;<br>Eye gaze;<br>GSR;<br>Respiration;<br>Skin temperature;<br>ECG;<br>Face/body videos	27	Dimensional (S):<br>Valence,<br>Arousal,<br>Dominance,<br>Predictability;<br>Discrete (S):<br>9 labels	Movies excerpts	-
Belfast<br>(Sneddon et al., 2011)	Videos;<br>Speech	114(T1)<br>82(T2)<br>60(T3)	Discrete (S/E):<br>8 labels	3 different<br>induction tasks	
SEMAINE<br>(McKeown et al., 2011)	Face videos;<br>Speech	150	Dimensional (E):<br>Valence, Arousal	Conversations<br>with an agent	FACS annotations<br>(?)
FER2013<br>(Goodfellow et al., 2013)	Images from web<br>queries	-	Discrete (S):<br>7 labels	-	35,887 images
MPI Emotional<br>Body Expression<br>Database<br>(Volkova et al., 2014)	Motion Capture<br>Videos (23 joints)	8	Discrete (E):<br>11 labels	Acting iin<br>non-verbal<br>scenarios and<br>short sentences	-
EmoReact<br>(Nojavanasghari et al., 2016)	Videos	63<br>children	Dimensional (E):<br>Valence;<br>Discrete (E):<br>16 labels	-	-
ASCERTAIN<br>(Subramanian et al., 2016)	EEG;<br>GSR;<br>ECG;<br>Face videos;	58	Dimensional (S):<br>Valence, Arousal	Movies excerpts	Big-Five personality traits<br>annotations<br>(?)
AffectNet<br>(Mollahosseini et al., 2017)	Images from web<br>queries	-	Categorical (E)l:<br>11 labels;<br>Dimensional (E):<br>Valence,<br>Arousal	-	450,000 images
AMIGOS<br>(Correa et al., 2018)	EEG;<br>GSR;<br>ECG;<br>Face/body videos;<br>Body depth maps	40	Dimensional (S):<br>Valence, Arousal,<br>Dominance, Liking<br>Familiarity;<br>Dimensional (E):<br>Valence, Arousal;<br>Discrete (S):<br>7 labels	Movies excerpts	Individual and group sessions;<br>Big-Five personality traits<br>(?);<br>PANAS annotations<br>(?)
JL-Corpus<br>(James et al., 2018)	Speech	4	Discrete (E):<br>10 labels	Acting	New Zealand<br>English
CAER<br>(Lee et al., 2019)	Videoclips from<br>TV shows	-	Discrete (E):<br>7 labels	-	Context labeling<br>13,201 clips,<br>1,107,877 frames

Table S1: Datasets for emotion recognition.

REFERENCES

Brendel, M., Zaccarelli, R., and Devillers, L. (2010). Building a system for emotions detection from speech to control an affective avatar. In *LREC*

Burkhardt, F., Paeschke, A., Rolfes, M., Sendlmeier, W. F., and Weiss, B. (2005). A database of german emotional speech. In *Ninth European Conference on Speech Communication and Technology*

Busso, C., Bulut, M., Lee, C.-C., Kazemzadeh, A., Mower, E., Kim, S., et al. (2008). Iemocap: Interactive emotional dyadic motion capture database. *Language resources and evaluation* 42, 335

Castellano, G., Leite, I., Pereira, A., Martinho, C., Paiva, A., and McOwan, P. W. (2010). Inter-act: An affective and contextually rich multimodal video corpus for studying interaction with robots. In *Proceedings of the 18th ACM international conference on Multimedia* (ACM), 1031–1034

Correa, J. A. M., Abadi, M. K., Sebe, N., and Patras, I. (2018). Amigos: A dataset for affect, personality and mood research on individuals and groups. *IEEE Transactions on Affective Computing*

Gnjatovic, M. and Rosner, D. (2010). Inducing genuine emotions in simulated speech-based human-machine interaction: The nimitek corpus. *IEEE Transactions on Affective Computing* 1, 132–144

Goodfellow, I. J., Erhan, D., Carrier, P. L., Courville, A., Mirza, M., Hamner, B., et al. (2013). Challenges in representation learning: A report on three machine learning contests. In *International Conference on Neural Information Processing* (Springer), 117–124

James, J., Tian, L., and Watson, C. I. (2018). An open source emotional speech corpus for human robot interaction applications. In *INTERSPEECH*. 2768–2772

Koelstra, S., Muhl, C., Soleymani, M., Lee, J.-S., Yazdani, A., Ebrahimi, T., et al. (2011). Deap: A database for emotion analysis; using physiological signals. *IEEE transactions on affective computing* 3, 18–31

Lee, J., Kim, S., Kim, S., Park, J., and Sohn, K. (2019). Context-aware emotion recognition networks. In *Proceedings of the IEEE International Conference on Computer Vision*. 10143–10152

Lucey, P., Cohn, J. F., Kanade, T., Saragih, J., Ambadar, Z., and Matthews, I. (2010). The extended cohn-kanade dataset (ck+): A complete dataset for action unit and emotion-specified expression. In *2010 ieee computer society conference on computer vision and pattern recognition-workshops* (IEEE), 94–101

Lundqvist, D., Flykt, A., and Öhman, A. (1998). The karolinska directed emotional faces (kdef). *CD ROM from Department of Clinical Neuroscience, Psychology section, Karolinska Institutet* 91, 2–2

Lyons, M., Akamatsu, S., Kamachi, M., and Gyoba, J. (1998). Coding facial expressions with gabor wavelets. In *Proceedings Third IEEE international conference on automatic face and gesture recognition* (IEEE), 200–205

McKeown, G., Valstar, M., Cowie, R., Pantic, M., and Schroder, M. (2011). The semaine database: Annotated multimodal records of emotionally colored conversations between a person and a limited agent. *IEEE transactions on affective computing* 3, 5–17

Mollahosseini, A., Hasani, B., and Mahoor, M. H. (2017). Affectnet: A database for facial expression, valence, and arousal computing in the wild. *IEEE Transactions on Affective Computing* 10, 18–31

Nojavanasghari, B., Baltrušaitis, T., Hughes, C. E., and Morency, L.-P. (2016). Emoreact: a multimodal approach and dataset for recognizing emotional responses in children. In *Proceedings of the 18th acm international conference on multimodal interaction*. 137–144

Rollet, N., Delaborde, A., and Devillers, L. (2009). Protocol cinemo: The use of fiction for collecting emotional data in naturalistic controlled oriented context. In *2009 3rd International Conference on Affective Computing and Intelligent Interaction and Workshops* (IEEE), 1–6

Savran, A., Alyüz, N., Dibeklioğlu, H., Çeliktutan, O., Gökberk, B., Sankur, B., et al. (2008). Bosphorus database for 3d face analysis. In *European Workshop on Biometrics and Identity Management* (Springer),

47–56

Sneddon, I., McRorie, M., McKeown, G., and Hanratty, J. (2011). The belfast induced natural emotion database. *IEEE Transactions on Affective Computing* 3, 32–41

Soleymani, M., Lichtenauer, J., Pun, T., and Pantic, M. (2011). A multimodal database for affect recognition and implicit tagging. *IEEE transactions on affective computing* 3, 42–55

Subramanian, R., Wache, J., Abadi, M. K., Vieriu, R. L., Winkler, S., and Sebe, N. (2016). Ascertain: Emotion and personality recognition using commercial sensors. *IEEE Transactions on Affective Computing* 9, 147–160

Volkova, E., De La Rosa, S., Bülthoff, H. H., and Mohler, B. (2014). The mpi emotional body expressions database for narrative scenarios. *PloS one* 9

Wang, S., Liu, Z., Lv, S., Lv, Y., Wu, G., Peng, P., et al. (2010). A natural visible and infrared facial expression database for expression recognition and emotion inference. *IEEE Transactions on Multimedia* 12, 682–691

Yin, L., Wei, X., Sun, Y., Wang, J., and Rosato, M. J. (2006). A 3d facial expression database for facial behavior research. In *7th international conference on automatic face and gesture recognition (FGR06)* (IEEE), 211–216
